# Shock index and TIMI risk index as valuable prognostic tools in patients with acute coronary syndrome complicated by cardiogenic shock

**DOI:** 10.1371/journal.pone.0227374

**Published:** 2020-01-03

**Authors:** Karolina Supeł, Michał Kacprzak, Marzenna Zielińska

**Affiliations:** Intensive Cardiac Therapy Clinic, Medical University of Lodz, Lodz, Poland; University of Tampere, FINLAND

## Abstract

**Background:**

The aim of the study was to evaluate the usefulness of the shock index (SI) and the TIMI risk index (TRI Thrombolysis in Myocardial Infarction Risk Index) one hour after successful primary percutaneous coronary intervention (pPCI) for predicting in-hospital mortality in patients with acute coronary syndrome complicated by cardiogenic shock (CS).

**Methods:**

Forty-seven consecutive patients with acute myocardial infarction (AMI) complicated by CS were included in this prospective observational study. All patients underwent pPCI and obtained TIMI Grade Flow 3. SI and TRI were calculated one hour after pPCI.

**Results:**

The primary endpoint—death from cardiovascular causes—occurred in 17 patients (36%). All calculated parameters were significantly higher in fatal CS than in the non-fatal CS group. A multivariate logistic regression model found only TRI to be an independent, significant predictor of death in the study group, with a proposed cut-off point of 66, with sensitivity 76.5% and specificity 83.3% (AUC 0.811, p = 0.00001).

**Conclusions:**

The simple parameters of clinical assessment—SI and TRI—calculated one hour after a successful pPCI of infarct related artery are important predictors of death in AMI complicated by cardiogenic shock.

## Introduction

In recent years, the mortality rate for AMI patients has been significantly reduced due to a key therapeutic strategy—early revascularization. Despite this, mortality in AMI complicated by cardiogenic shock has remained at the same high level [1]. Even after successful revascularization, it is impossible to confidently state whether a patient with cardiogenic shock in the course of ACS will survive. Therefore, there is a need for a simple and fast tool that promptly identifies patients with the highest risk of death among those recovering from successful PCI of infarct related artery (IRA) with ongoing cardiogenic shock. Both the shock index (SI), a ratio of heart rate/systolic blood pressure, and TIMI risk index (TRI) calculated using the equation [heart rate x (age/10)^2^/systolic blood pressure] appear to be suitable candidates. SI was first introduced to evaluate the degree of hypovolemia in hemorrhagic and infectious shock states by Allgower and Burri [2]; then, it was widely used in critically-ill patients and served as an indicator of severity of a disease [3,4]; currently, it is used for the prediction of mortality in ACS populations. TRI was derived from the InTIME II substudy, a strong and independent predictor of 30-day mortality and a robust predictor of a very early events, including death within 24 hours in STEMI [5].

The aim of our study was to evaluate the potential of SI and the TRI, both calculated one hour after primary PCI, for predicting in-hospital mortality in patients with acute coronary syndrome (ACS) complicated by cardiogenic shock (CS).

## Materials and methods

This prospective observational study included 47 consecutive patients admitted to the Intensive Cardiac Therapy Clinic. All were aged from 37 to 91 years, and presented with AMI complicated by cardiogenic shock, admitted to the Intensive Cardiac Therapy Clinic were included in our prospective observational study.

AMI was diagnosed in accordance with the Third Universal Definition of Myocardial Infarction [6]. The diagnosis of cardiogenic shock was based on a clinical presentation including systolic blood pressure < 90 mmHg, pulmonary congestion and symptoms of low cardiac output: sinus tachycardia, renal dysfunction and reduced urinary output, altered mental status, signs of poor peripheral perfusion [1]. Exclusion criteria were reversible causes of low blood pressure, the mechanical complications of myocardial infarction (acute mitral valve regurgitation, cardiac rupture, ventricular septal rupture, cardiac tamponade) and necessity of urgent coronary artery bypass grafting.

Demographic, clinical, biochemical, ECG and echocardiographic data were collected on admission in all patients. Vital signs, clinical status and routine laboratory parameters were assessed each day. The vital signs after PCI were measured one hour after the procedure in the supine position. SI was defined as the ratio of HR and SBP [5]. The TRI was calculated using the equation: [heart rate x (age/10)^2^/systolic blood pressure] [7]. The optimal cut-off value of SI and TRI was based on receiver operating characteristic curve analysis.

Electrical instability was defined as occurrence of ventricular tachycardia (VT), ventricular fibrillation (VF) or atrioventricular block (AVB) within the first 24 hours of hospitalization.

Screening transthoracic echocardiography was performed to rule out mechanical complications of AMI and other acute cardiac diseases with chest pain and hypotension (acute aortic dissection or pulmonary embolism) and to estimate left ventricular ejection fraction (EF).

All patients underwent urgent coronary angiography and primary percutaneous coronary intervention (PCI). Infarct related artery (IRA) was treated during the intervention; 43 patients (91.5%) underwent coronary drug-eluting stent implantation (DES) the remaining four (8.5%) underwent conventional balloon angioplasty. Patients with multiple, critical stenoses were subjected to a complete revascularization during the prompt intervention (n = 11, 26%). All patients achieved TIMI Grade Flow 3. According to the operator’s decision, 22 patients (51%) received GPIIb/IIIa inhibitor (abciximab).

All patients received intravenous infusion of catecholamines to maintain organ perfusion. Mechanical support (IABP—intra- aortic balloon pumping) was used in 19 patients (40%) who did not achieve a satisfactory increase in blood pressure despite intravenous infusion of inotropic agents.

The primary endpoint was death from cardiovascular causes.

### Statistical analysis

Categorical variables were summarized as frequencies with percentage values. The Shapiro–Wilk test was used to determine whether a variable followed a normal distribution. Non-parametric statistics were used for variables with a non-normal distribution. Continuous variables with normal distribution were expressed as means ± standard deviation (SD), and those with a non-normal distributions as medians with interquartile range. Due to the small group size and lack of normal distribution in some of the variables, quantitative variables are presented as median with interquartile range. Correlations were assessed using the Spearman's rank correlation coefficient. Differences between continuous variables were compared with the Student’s T-test, Mann–Whitney U test or with the ANOVA and Kruskall–Wallis test depending on the variable. Categorical variables were compared with the chi-squared test with Yates's correction for continuity. To assess the relationship between one or more independent variables and a dichotomous dependent variable (i.e. occurrence of death), backward stepwise logistic regression was used. To assess the suitability of TRI and SI levels in predicting adverse cardiac events ROC curves were used. All statistical analyses were performed using Statistica 12.5 (StatSoft Inc., USA). P value <0.05 were considered statistically significant.

The study protocol was approved by the local ethics committee (Bioethics Committee at the Medical University of Lodz, Poland) and followed the guidelines of the Declaration of Helsinki (RNN/75/11/KE 2011). When feasible, written informed consent was obtained before enrolment by conscious patients. In other cases, patients‘ next of kin signed the agreement, which was confirmed by the patient after regaining consciousness.

## Results

### Patient characteristic

Forty-seven patients (19 women [40%] and 28 men [60%]) were included in this study. Their median age was 68 ((Q1; Q3 64; 71); range 37–91) years. All patients demonstrated symptoms of acute myocardial infarction and cardiogenic shock on admission. The baseline characteristics of patients are summarized in Table 1. All patients underwent urgent coronary angiography and PCI of IRA ending with TIMI Grade Flow 3. Multi-vessel disease was observed in 35 patients (75%). Eleven patients (26%) required complete revascularization during the prompt intervention. Coronary angiography findings and details of invasive treatment are presented in Table 2. Full echocardiography was performed on subsequent days of hospitalization in 36 patients (77%). The median EF was 40 (Q1; Q3 32,49)%. Left ventricular systolic function was assessed as preserved or moderately impaired (EF > 40%) in 16 patients (44%) and significantly impaired (EF ≤ 40%) in 20 patients (56%). All patients received intravenous infusion of catecholamines: in 19 patients (40%) IABP was applied for a mean duration of four days. No significant difference was observed between the group receiving mechanical support and the group without with regard to the occurrence of in-hospital mortality (p = 0.339).

**Table 1:** Baseline characteristics of the study group.

**Type of MI**	**n**	**%**
Antero-lateral STEMI	17	36
Infero-posterior STEMI	17	36
New LBBB	2	4
NSTEMI	11	23
Right ventricle infarction	10	21
**Cardiovascular risk factors**	n	%
Hypertension	27	58
Diabetes mellitus	12	26
Active smokers	16	34
History of MI	15	32
**Parameter (units) on admission**	**Median**	**(Q1; Q3)**
Creatinine (umol/l)	111	(94; 136)
GFR (ml/min/1,73m^2^)	49	(37; 63)
ALAT (U/l)	41,5	(23; 156)
ASPAT(U/l)	111	(40; 184)
CRP hs (mg/l)	7,7	(2.5; 22.6)
Electrical instability	n	%
VF/VT acute	21	45
AVB	11	23
**Other**	**Median**	**(Q1; Q3)**
SBP (mmHg)	90	(70; 100)
HR (beat/min)	94	(80; 110)
SI	1,1	(0.9; 1.4)
TRI	48	(31; 79)
B-blocker before admission (n; %)	18	38

MI, myocardial infarct; STEMI, ST-segment elevation myocardial infarction; LBBB, left bundle branch block; NSTEMI, non-ST-elevation myocardial infarction, GFR, glomerular filtration rate; VF, ventricular fibrillation; VT, ventricular tachycardia; AVB, atrioventricular block; SBP systolic blood pressure; HR, heart rate; SI, Shock Index; TRI, TIMI Risk Index.

**Table 2:** Coronarography findings and details of invasive treatment.

	n	%
Multi-vessel CAD	35	75
Singlevessel CAD	12	25
PCI with DES	43	91,5
POBA	4	8,5
PCI > 1 vessel	11	26
GPIIb/IIIa inhibitor	22	51
IABP	19	40

CAD, coronary artery disease; PCI, percutaneous coronary intervention; POBA, percutaneous balloon angioplasty; IABP, intra-aortic balloon pump.

The primary endpoint i.e. death from cardiovascular causes occurred in 17 patients (36%). Eight patients (17%) died on the first day of hospitalization.

### SI and TRI after primary percutaneous coronary intervention

One hour after PCI, the mean HR was 93 ± SD 24 beat/min, mean SBP 82 ± SD 23 mmHg, SI 1.1 (Q1; Q3 0.9; 1.4) and TRI was 48 (Q1; Q3 31; 79). None of the parameter values differed significantly with regard to gender, the occurrence of diabetes or electrical instability. TRI was significantly higher in patients with hypertension (p = 0.015) and in patients who were administered b-blocker before hospitalization (p = 0.0039). SI and TRI were significantly higher in patients with the history of myocardial infarction (p = 0.0298, 0.0037 respectively).

**Fig 1:**
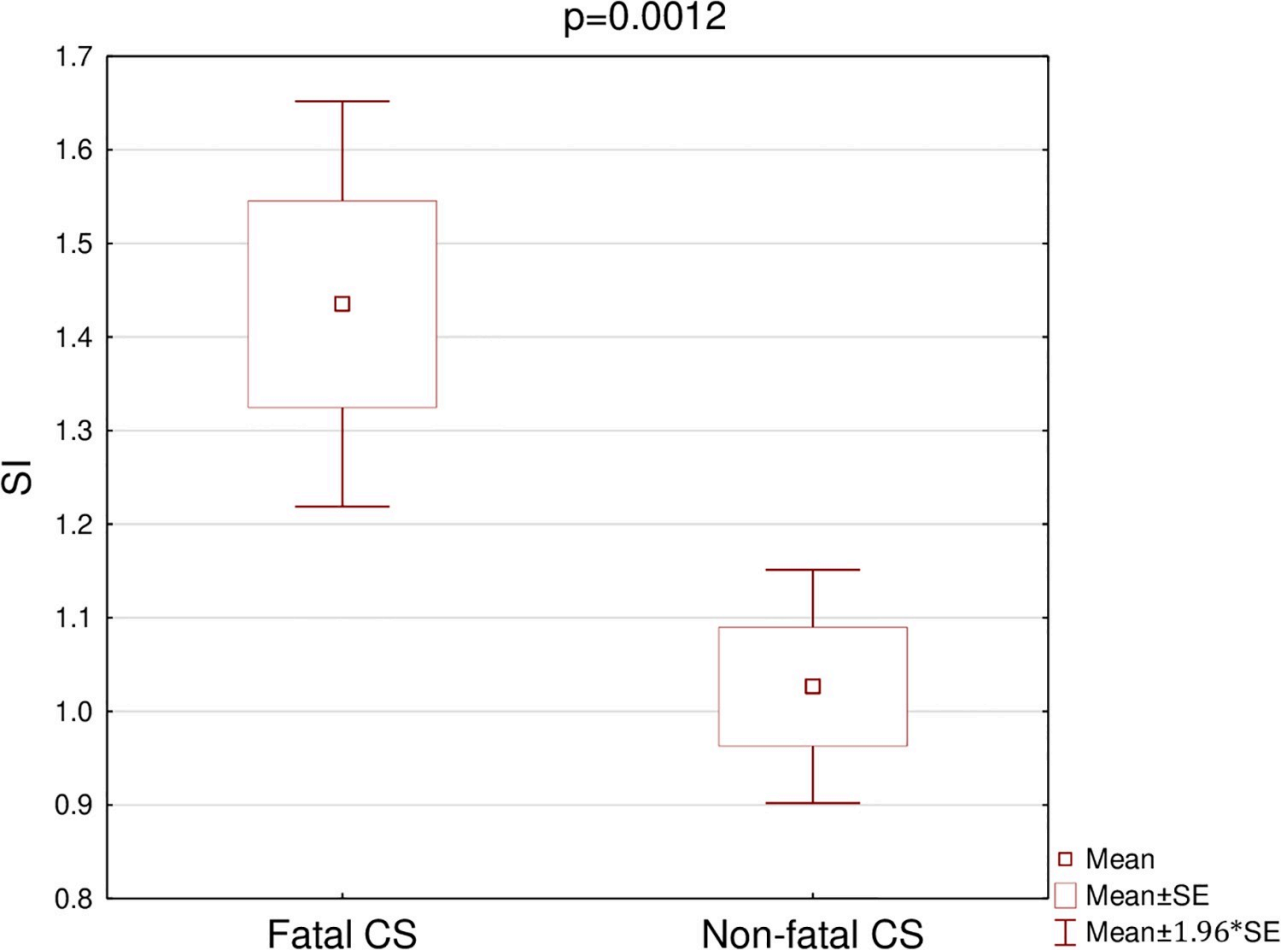
Comparison of SI in patients with fatal and non-fatal cardiogenic shock. SI, Shock Index; Fatal CS, Fatal cardiogenic shock; Non-fatal CS, Non-fatal cardiogenic shock.

**Fig 2:**
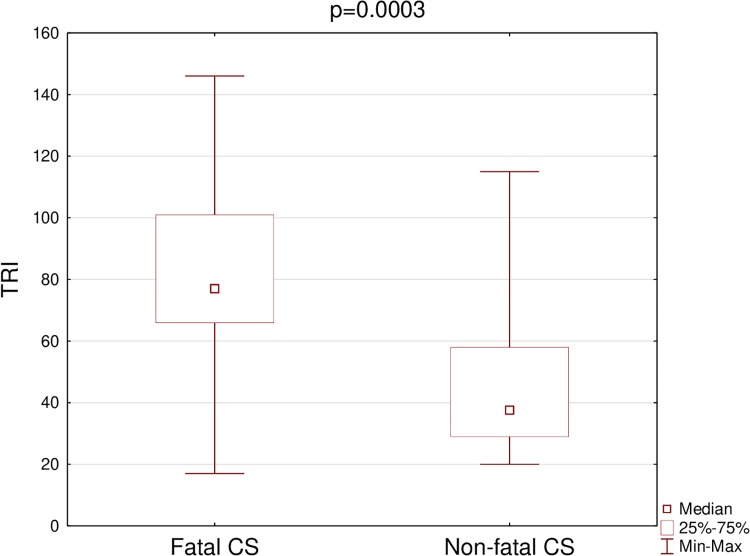
Comparison of TRI in patients with fatal and non-fatal cardiogenic shock. TRI, TIMI Risk Index; Fatal CS, Fatal cardiogenic shock; Non-fatal CS, Non-fatal cardiogenic shock.

Both SI and TRI were significantly higher in the fatal CS than in the non-fatal CS group (Table 3) (Figs 1 and 2). The cut-off point was 1.1 for SI, and 66 for TRI. For SI 60% mortality was observed above the cut-off (i.e. ≥ 1.1) and 9,1% below it (i.e. < 1.1) (p = 0.0009). In addition 72.2% mortality was observed for TRI ≥ 66 and 13.8% for TRI < 66 (p = 0.0002).

**Table 3:** Parameters and their p—value in fatal vs non-fatal cardiogenic shock.

Variable	Fatal vs non-fatal CS	p
SI	1.4 vs 1.03	**0,0012**
TRI	77 vs 37.5	**0,0003**

CS, cardiogenic shock; SI, Shock Index; TRI, TIMI Risk Index.

**Fig 3:**
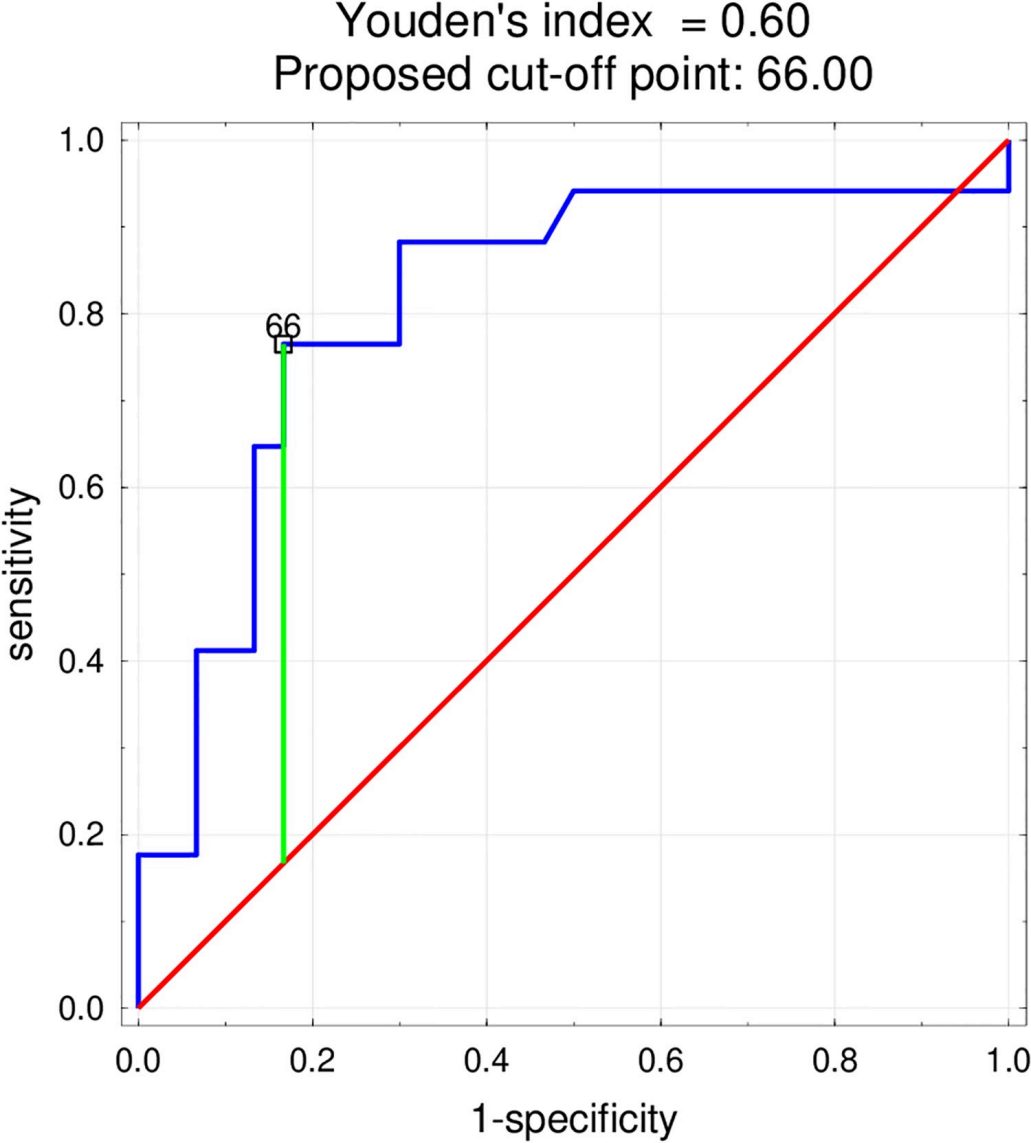
The ROC curve—Variable tested: TRI as a predictor of death in myocardial infarction complicated by cardiogenic shock.

Univariate logistic regression and ROC curves were used to compare potential predictors of death. SI, TRI, age, sex, diabetes, electrical instability, pulmonary oedema, creatinine, haemoglobin and glycaemia on admission were included in the multivariate logistic regression model. Of the all parameters, only TRI was an independent and significant predictor of death with the proposed cut-off point 66, with sensitivity 76.5% and specificity 83.3% in the study group (p = 0.0019, OR 1.039, CI ±95%: 1.014–1.065; AUC 0.811, p = 0,00001) (Fig 3). The model was validated by the k-fold cross-validation method and the obtained AUC value was 0.800.

## Discussion

Our findings confirm that SI and TRI calculated one hour after successful pPCI are valuable prognostic tools in patients with AMI complicated by cardiogenic shock: both SI and TRI were significantly higher in patients who died.

Previous studies have used SI to predict mortality in ACS populations. It has been reported that elevated SI predicted short-term mortality in patients with STEMI [4, 8, 9] and NSTEMI [10]. A study conducted by Bilkova et al. showed that SI ≥ 0.8 on admission was associated with 20% mortality, whereas SI < 0.8 was associated with 4% mortality in 644 patients with STEMI (p < 0.01) [8]. In a study conducted by Huang et al., among 7 187 patients with STEMI, the in-hospital mortality rate on admission was 16% among patients who presented with SI > 0.7, and 4.6% among those demonstrating SI < 0.7 (p < 0.001) [4]. Kobayashi et al. report that SI ≥ 0.7 was associated with 4,9% mortality and SI < 0.7 with 0.5% mortality among 481 NSTEMI patients (p = 0.006) [10]. In our study, in patients with acute myocardial infarction complicated by cardiogenic shock those who presented SI ≥ 1.1 were associated with 60% mortality and those with SI < 1.1 presented 9.1% (p = 0.0009).

The InTIME II substudy found TRI to be a strong and independent predictor of mortality (c statistic = 0.78, p < 0.0001)—categorised into quintiles, revealed a more than 20-fold gradient of increasing mortality from 0.8% to 17.4% (p < 0.0001). Not only did TRI show an excellent concordance between the observed 30-day mortality but also was found to be a robust predictor of very early events, including death within 24 hours (c statistic = 0.81) [5]. Similarly, in a study carried out by Wiviott et al., TRI demonstrated a strong graded relationship with in-hospital mortality across the risk index categories among patients with STEMI, both those treated with reperfusion therapy or those without (c statistic 0.81; 0.71 respectively). The same relationship between the TRI and mortality was observed among patients with NSTEMI, with a > 30-fold difference in mortality rates between lowest and highest deciles (p < 0.0001, c statistic 0.73) [11]. Additionally, TRI has been found to offer promise for the prediction of mortality risk across the whole spectrum of acute coronary syndromes [12] and in a cohort of STEMI and non-STEMI patients [13]. It was found that the risk of 30-day mortality increased in the whole tested group by 6% for each point of the TIMI risk index [13]. Furthermore, TRI predicted increased long-term mortality and CHF in patients with STEMI in the TIMI 2 clinical trial: the median follow-up was three years, the group with the highest TRI level demonstrated more than 5-fold higher mortality and more than 4-fold higher risk of CHF [14].

In our study, multivariate logistic regression adjustment found TRI to be an independent and significant predictor of in-hospital mortality with a proposed cut-off point of 66; this approach demonstrated a sensitivity of 76.5% and specificity of 83.3% in the study group (AUC 0.800, p = 0,00001). TRI ≥ 66 was associated with 72.2% mortality, and TRI < 66 with 13.8% mortality. Interestingly the magnitude of myocardial damage measured by EF did not correlate with SI (p = 0.386) or TRI (p = 0.0536).

Our findings confirm that SI and TRI are also valuable prognostic tools in patients with cardiogenic shock as previously observed in those with ACS but without any such a complication. Mortality remains uncertain in patients with ongoing cardiogenic shock following successful revascularization of IRA in AMI. Fortunately, risk stratification can be made more effective by adopting a strategy based on SI and TRI, which can be easily acquired by evaluating readily-accessible clinical parameters.

### Study limitations

The small size of the study group and the fact it was conducted only in one center are the most important limitations. Another limitation is the occurrence of electrical instability and conduction abnormalities (AVB) as a complication of myocardial infarction. In addition, some patients were administered medication treatment before hospitalization, which might have influenced the heart rate and blood pressure (b-blocker intake 38%).

## Conclusions

SI and TRI calculated one hour after a successful pPCI of an infarct-related artery are important predictors of death in AMI complicated by cardiogenic shock.

## Supporting information

S1 Table Measured parameters and their values in the study group. F, female; SBP, systolic blood pressure; DBP, diastolic blood pressure; HR, heart rate; SI, Shock Index; TRI, TIMI Risk Index; HA, hypertension; DM 2, diabetes mellitus type 2.
(XLSX)
